# Reply to the letter regarding the article entitled: “Access to rehabilitation after stroke in Brazil (AReA study): multicenter study protocol”

**DOI:** 10.1055/s-0043-1771266

**Published:** 2023-07-26

**Authors:** Roberta de Oliveira Cacho, Carla Heloisa Cabral Moro, Rodrigo Bazan, Suzete Nascimento Farias da Guarda, Elen Beatriz Pinto, Suellen Mary Marinho dos Santos Andrade, Lenise Valler, Kelson James Almeida, Tatiana Souza Ribeiro, Renata Viana Brígido de Moura Jucá, Cesar Minelli, Maria Elisa Pimentel Piemonte, Eric Homero Albuquerque Paschoal, Marco Túlio Araújo Pedatella, Octávio Marques Pontes-Neto, Ana Paula Fontana, Aline de Souza Pagnussat, Adriana Bastos Conforto, AReA Study Group

**Affiliations:** 1 Universidade Federal do Rio Grande do Norte, Faculdade de Ciências da Saúde do Trairi, Santa Cruz RN, Brazil.; 2 Hospital Municipal de São José, Joinville SC, Brazil.; 3 Universidade Estadual Paulista, Faculdade de Medicina de Botucatu, São Paulo SP, Brazil.; 4 Universidade Federal da Bahia, Faculdade de Medicina da Bahia, Departamento de Neurociências e Saúde Mental, Salvador BA, Brazil.; 5 Escola Bahiana de Medicina e Saúde Pública, Fundação para o Desenvolvimento das Ciências, Salvador BA, Brazil.; 6 Universidade Federal da Paraíba, João Pessoa PB, Brazil.; 7 Universidade Federal de Campinas, Campinas SP, Brazil.; 8 Universidade Federal do Piauí, Centro Universitário UniFacid, Teresina PI, Brazil.; 9 Universidade Federal do Rio Grande do Norte, Natal RN, Brazil.; 10 Hospital Universitário Walter Cantídio, Fortaleza CE, Brazil.; 11 Hospital Carlos Fernando Malzoni, Instituto “Você sem AVC,” Matão SP, Brazil.; 12 Universidade de São Paulo, Faculdade de Medicina de Ribeirão Preto, Departamento de Neurociências e Ciências Comportamentais, Ribeirão Preto SP, Brazil.; 13 Universidade de São Paulo, Faculdade de Medicina, São Paulo SP, Brazil.; 14 Hospital Ophir Loyola, Belém PA, Brazil.; 15 Hospital Estadual de Urgência de Goiânia Dr Valdemiro Cruz, Goiânia GO, Brazil.; 16 Universidade de São Paulo, Faculdade de Medicina de Ribeirão Preto, Ribeirão Preto SP, Brazil.; 17 Universidade Federal do Rio de Janeiro, Faculdade de Fisioterapia, Laboratório Pesquisa em Recuperação Funcional Após AVC, Rio de Janeiro RJ, Brazil.; 18 Universidade Federal de Ciências da Saúde de Porto Alegre, Departamento de Fisioterapia, Porto Alegre RS, Brazil.; 19 Universidade de São Paulo, Hospital de Clínicas, São Paulo SP, Brazil.; 20 Hospital Israelita Albert Einstein, São Paulo SP, Brazil.

Letter to “Access to rehabilitation after stroke in Brazil (AReA study): multicenter study protocol”

Dear editor,

We appreciate Montanaro's 1 interest in our article “Access to rehabilitation after stroke in Brazil (AReA study): multicenter study protocol” 2 and the opportunity to emphasize the goal of the AReA study, to investigate access to post-stroke rehabilitation within the first 6 months after discharge from public hospitals. 2 Access is defined as the opportunity to utilize healthcare services at the ideal time for the rehabilitation process, regardless of an individual's needs. 3

A recent review of stroke guidelines for the care of people with stroke conducted by the World Stroke Organization Guideline Committee highlighted the need of rehabilitation provided not only by multidisciplinary but also by interdisciplinary teams, to provide comprehensive care for people with stroke. The teams should offer coordinated care, staffed by physicians, nurses, physiotherapists, occupational therapists, speech-language therapists, social workers, and nutritionists with expertise in stroke rehabilitation, recovery, and return to work. 4 As stated in our manuscript, we absolutely agree with this approach: “current guidelines recommended that subjects with stroke should be assessed by a multidisciplinary team within 24 to 48 hours after admission and referred to a rehabilitation program immediately after discharge.” 5

For this reason, we included questions about referrals to physical therapists, physiatrists, speech therapists, neurologists, psychologists, occupational therapies, and nutritionists (Table 1). 2 The first five questions are repeated in regard to referrals for each of these professionals, as stated in the line highlighted in gray, below the fifth question. In addition, the kind of rehabilitation service is asked in question 6. All responses will be collected and analyzed. We expect that the results of the AReA study will contribute to the situational understanding about access to multidisciplinary care in Brazil, which may be useful to guide public health policies to reduce the general and specific barriers to this kind of care in different Brazilian regions. We think that the participation of 22 centers distributed by five Brazilian regions is one of the main strengths of the AReA Study.

We also agree about the advantages of including tools to properly assess functionality in all its domains. Convinced about the central role of functionality as the primary outcome of the rehabilitation process, the AReA research group extensively discussed tool selection during the design phase of this protocol. Considering the (1) time for application of multiples tools that demand training for proper and standardizing application, (2) limitation of physical space and resources, and (3) the aim of the present study, we decided to include two validated tools: the NIH Stroke Scale, a standardized scale that provides assessment of neurological impairments, and the modified Rankin Scale that addresses disability will be used to characterize the patients included in the study. 6

Detailed evaluation of the *impact* of stroke on metrics of body function and structure, activity and participation according to the International Classification of Functioning, Disability and Health (ICF) 7 would certainly be part of another important study, with a different research question, demanding other design and methods. The protocol of such a study would require an increased length in the duration of interviews and physical examinations, compared with the AReA protocol. We expect that results from the present protocol will be able to guide the design of new studies in this field. Importantly, we also hope that results from the AReA study provide an alert to the relevance of studies in this field, and the need of funding for this kind of research.

Barriers to inclusion in a comprehensive study about the *impact* of stroke, such as difficulties in accessible transportation for patients with disabilities and the need of physical help from caregivers to attend sessions, may lead to bias if patients who can comply with the protocol schedule are also those with greater social support and economic conditions. For instance, caregivers may be available to accompany individuals with stroke to attend outpatient consults that include short interviews, but not long assessments. This, and other limitations for participation in rehabilitation studies have been documented by several studies in Brazil and in other countries. 8 Advances in digital health may contribute to overcome physical barriers but will also request access to the internet and ability to communicate by means of computers or smartphones. We suggest that these variables be taken into consideration during the design of future studies about the *impact* of stroke.
